# Lead Exposure in Newly Resettled Pediatric Refugees in Syracuse, NY

**DOI:** 10.1007/s10903-019-00880-y

**Published:** 2019-03-20

**Authors:** Christina D. Lupone, Danielle Daniels, Dawn Lammert, Robyn Borsuk, Travis Hobart, Sandra Lane, Andrea Shaw

**Affiliations:** 1 Department of Public Health & Preventive Medicine, SUNY Upstate Medical University, 750 East Adams Street, Syracuse, NY 13210 USA; 2 Institute for Global Health and Translational Science, SUNY Upstate Medical University, Syracuse, NY USA; 3 Department of Pediatrics, SUNY Upstate Medical University, Syracuse, NY USA; 4 College of Medicine, SUNY Upstate Medical University, Syracuse, NY USA; 5 Falk College, Syracuse University, Syracuse, NY USA; 6 Department of Obstetrics and Gynecology, SUNY Upstate Medical University, Syracuse, NY USA

**Keywords:** Refugee, Lead exposure, Pediatric, Anemia, Environmental inequalities

## Abstract

Lead is a major environmental toxin that presents numerous health consequences for children. Refugee children are at a risk of lead poisoning post-resettlement due to urban housing and environmental inequalities stemming from lack of funding, legislation, and advocacy. This article addresses lead exposure upon arrival and post-resettlement in 705 refugee children (age 0–16 years) attending a university clinic in Syracuse, NY, a city with a large refugee population. 17% of the newly arrived children had elevated blood lead levels (BLLs) (≥ 5 µg/dL); 10% had elevated BLL upon follow-up; 8.3% of the children’s follow-up elevated BLL were new exposures. 30% were found to have increased BLL at follow-up regardless of arrival status. An analysis of new exposures found a significant proportion of children would have been missed on routine screening that targets children < 2 years old. Primary prevention efforts are needed to prevent exposure and address risks to improve the health of all children locally, including newly resettled refugees.

## Background

By the end of 2016, the United Nations High Commission for Refugees (UNHCR) estimated that 65.6 million people were forcibly displaced from their homes; 22.5 million requested refugee status, with over half of them being children [1]. New York State was ranked 5th highest in the United States (US) for refugee resettlement in 2017 [2], with Onondaga county ranked 2rd highest per capita for refugees resettled in the state [3]. Over the past decade, refugees have arrived in Syracuse from countries spread across the globe, including Africa (Somalia, Democratic Republic of Congo, Sudan, South Sudan, Central African Republic, Eritrea, Burundi), Asia (Bhutan, Myanmar), Eastern Europe (Russia, Ukraine), and the Middle East (Syria, Afghanistan, Iraq) [4].

## Lead Screening Recommendations

Due to the wide variation of lead exposure within communities across the United States, the Centers for Disease Control and Prevention (CDC) and the American Academy of Pediatrics recommend individualized lead screening guidelines based on local data [5]. New York State regulations require testing at ages 1 and 2 years with regular assessment of risk factors for lead exposure (e.g. peeling paint exposure, parental employment involving lead exposure, sibling with lead exposure, pica-like behavior) until the age of 6 [6]. Both New York State and CDC guidelines recommend routine blood lead level (BLL) screening and nutritional evaluations for refugee children from birth to 16 years upon arrival to the US; a second BLL is recommended 3–6 months after they establish permanent residence regardless of initial screening results [7, 8]. BLL’s < 5 µg/dL are considered acceptable or below reference level; results of 5 µg/dL and higher are clinically significant and warrant increasing levels of intervention including nutritional counseling, environmental assessments and investigations, lab work, neurological exams, and if warranted, chelation therapy [9]. Impediments to accurately screening refugee children include language barriers [10], cross-cultural misunderstandings, low health literacy [11], and increased vulnerability to inadequate healthcare [12].

## Lead Exposure in Refugee Children

The prevalence of elevated BLLs in refugee children is double that of US born children [13]. Refugee children often experience high lead environments in their home countries, as well as refugee camps and other living situations through which they pass during their flight to safety [14, 15]. Overseas sources of lead include gasoline, lead-acid battery recycling, and mining. Household exposures include burning lead-containing materials (paper products, discarded rubber, painted wood) for cooking and heating, glazed ceramic cookware, traditional remedies, food supplements, and cosmetics [16, 17].

The primary risk factor for lead exposure in newly resettled refugee children in the US is urban housing built before 1978, when lead was banned from house paint [18]. In Syracuse, NY most refugees are resettled in older rental houses with risk of lead exposure. A cohort study of refugees settled in Colorado, Minnesota, Philadelphia and Washington State found between 1–3% had elevated BLLs (≥ 10 µg/dL) on arrival to the US [19]. Another cohort study in Massachusetts found 11.3% of recently arrived refugee children had blood lead levels ≥ 10 µg/dL, while 7% of the cohort with available testing ≥ 6 months after arrival had newly elevated levels suggestive of new exposures [13]. Although the overall incidence of elevated BLL in the US is decreasing [20], environmental inequality persists in the impoverished communities where refugees are resettled and children are at high risk of lead poisoning [14, 21–23]. This same environmental inequality exists within the city of Syracuse where the burden of lead exposure remains significant for all children regardless of refugee status [24].

## Anemia and Lead Absorption

Iron deficiency anemia is a risk factor for lead toxicity, as it not only increases the incidence of pica behavior, but also enhances absorption of lead from the GI tract [25, 26]. According to the National Health and Nutrition Examination Survey of US children age 0–3 years, 9% have iron deficiency and 2% have iron deficiency anemia [26]. Lead exposure and nutritional deficiencies put children at risk for developmental delay and behavioral challenges [27].

## The Current Study

Our study describes lead levels of refugee children upon arrival and post- resettlement in Syracuse, NY. In addition to lead exposure, the study examines the relationship of iron deficiency anemia on arrival and the risk for elevated BLL post-resettlement [7].

## Methods

### Research Design, Participants, and Ethical Issues

The study used a cross-sectional retrospective chart analysis of all pediatric refugee patients (ages 0–16) attending the Pediatric Refugee Clinic at SUNY Upstate Medical University between May 31, 2012 and June 1, 2017. The study was granted an exemption from the SUNY Upstate Medical University Institutional Review Board. No unique patient-identifying information was linked with the data at the time of analysis.

### Data Collection

Electronic chart extraction was performed for all newly resettled refugee children age 0–16 years at the time of arrival. The chart review of the pediatric refugee patients’ intake evaluation within 90 days of their US arrival extracted complete blood count with differential, BLL obtained from venous blood draw, and free erythrocyte protoporphyrin (FEP) level. Variables included age (in years), number of children in the family, country of origin, country of refuge, number of years in country of refuge, year of arrival in the US, sex (male/female), red blood cell count (×10^6^/uL), hemoglobin level (g/dL), mean corpuscular volume (fL), red cell distribution width (%), and BLL at baseline and 6 month follow-up (µg/dL).

Inclusion criteria were refugee children aged 0–16 years upon arrival in the United States with established pediatric care at the University healthcare center. Children were excluded if they did not have a complete blood panel and BLL at baseline or 3–6 months post arrival (follow-up) recorded in their electronic medical record.

Data was analyzed using Microsoft Excel (Microsoft Corporation, Redmond, Wash, USA) and SPSS Version 22.0 (IBM, Chicago, IL, USA). Analyses included the calculation of frequencies, means, standard deviations, Pearson Chi-Square tests, and Fisher’s Exact tests. Results were considered significant if p ≤ 0.05. *P* values are reported as Pearson Chi-Square unless noted otherwise. Associations between BLL were analyzed in relation to participant demographics (sex, age, country of origin, country of refuge). Associations between BLL and relation to anemia (present yes/no) and Red Cell Distribution (RDWI) Width Index were completed. RDWI calculation = Mean Corpuscular Volume × Red Cell Distribution Width/Red Blood Cell Count. If calculation is > 220 results are consistent with iron deficiency anemia, if < 220, results are consistent with thalassemia trait. The datasets generated during and analyzed during the current study are available from the corresponding author on reasonable request.

## Results

### Descriptive Statistics

A total of 892 refugee children established as new refugee patients during the study period; 705 children from 278 families met inclusion criteria. Among the 187 children excluded, 185 did not obtain a follow up BLL. Of the remaining two children excluded, one did not have an initial BLL, and another did not have any blood work reported including CBC. Excluded children were 41.7% female with a mean age of 8.8 years (range 1–16 years at chart review) and accounted for 76 families. Of the excluded children with initial BLLs reported, 13.8% were found to have elevated (≥ 5.0 µg/dL). Many of these families left Syracuse to join family members in other parts of the US.

Children were 51.2% female with a mean age of 8.0 years (range 1–16 years at chart review). Children spent an average of 6.2 years in the country of refuge prior to resettlement (range of 1–16 years). Year of arrival in the US ranged from 2012 to 2017 with the largest number of resettlements occurring in 2016 (23.0%). Table 1 presents the countries of refuge by region prior to resettlement in the US. BLLs were reported as below the reference level (0–4.9 µg/dL), elevated (5.0–9.9 µg/dL), and highly elevated (≥ 10.0 µg/dL). Upon arrival, 585 (83.0%) of refugee children had a BLL below the reference level, with 108 (15.3%) and 12 (1.7%) of the children recording elevated and highly elevated blood lead levels, respectively. Children aged 0–6 years were more likely to have elevated BLL upon arrival than children aged 7–16 years (23.6% vs. 12.5%, respectively; *p* < 0.01).

**Table 1:** Demographics of country of refuge by region

Region of refuge	N (%)
Africa	375 (53.2)
Middle East	268 (38.0)
Southeast Asia	52 (7.4)
Eastern Europe	10 (1.4)

Countries represented in each region: Southeast Asia (Thailand, Nepal, Malaysia, Indonesia, Bhutan, China); Africa (Botswana, Burundi, Cameroon, Chad, Congo, Djibouti, Ethiopia, Kenya, Malawi, Morocco, Namibia, Rwanda, South Africa, Tanzania, Togo, Tunisia, Uganda); Eastern Europe (Malta, Romania, Slovakia, Ukraine) Middle East (Afghanistan, Egypt, Iraq, Jordan, Lebanon, Pakistan, Sudan, Syria, Turkey, Yemen)

A total of 278 families were included in the data extraction. 17.0% of families represented had at least one child with a BLL above the reference level upon arrival and 9.9% of families had at least one child with a BLL above the reference level at follow-up. Ten families had all children affected upon arrival; three families were found to have all children affected post-resettlement as new exposures.

Results by year of arrival found that clinically significant BLLs upon arrival and follow up continue to plague the community despite clinical and public health efforts. On average 17.0% of refugee children arrived each year with clinically significant BLLs (range 13.6–20.3%) and 9.9% on average (range 7.1–18.2%) of children were elevated upon follow up. The proportion of children remaining at risk with clinically significant BLLs at follow up has increased from 2014 to 2017 from 7.1 to 18.2%, respectively.

**Fig. 1:**
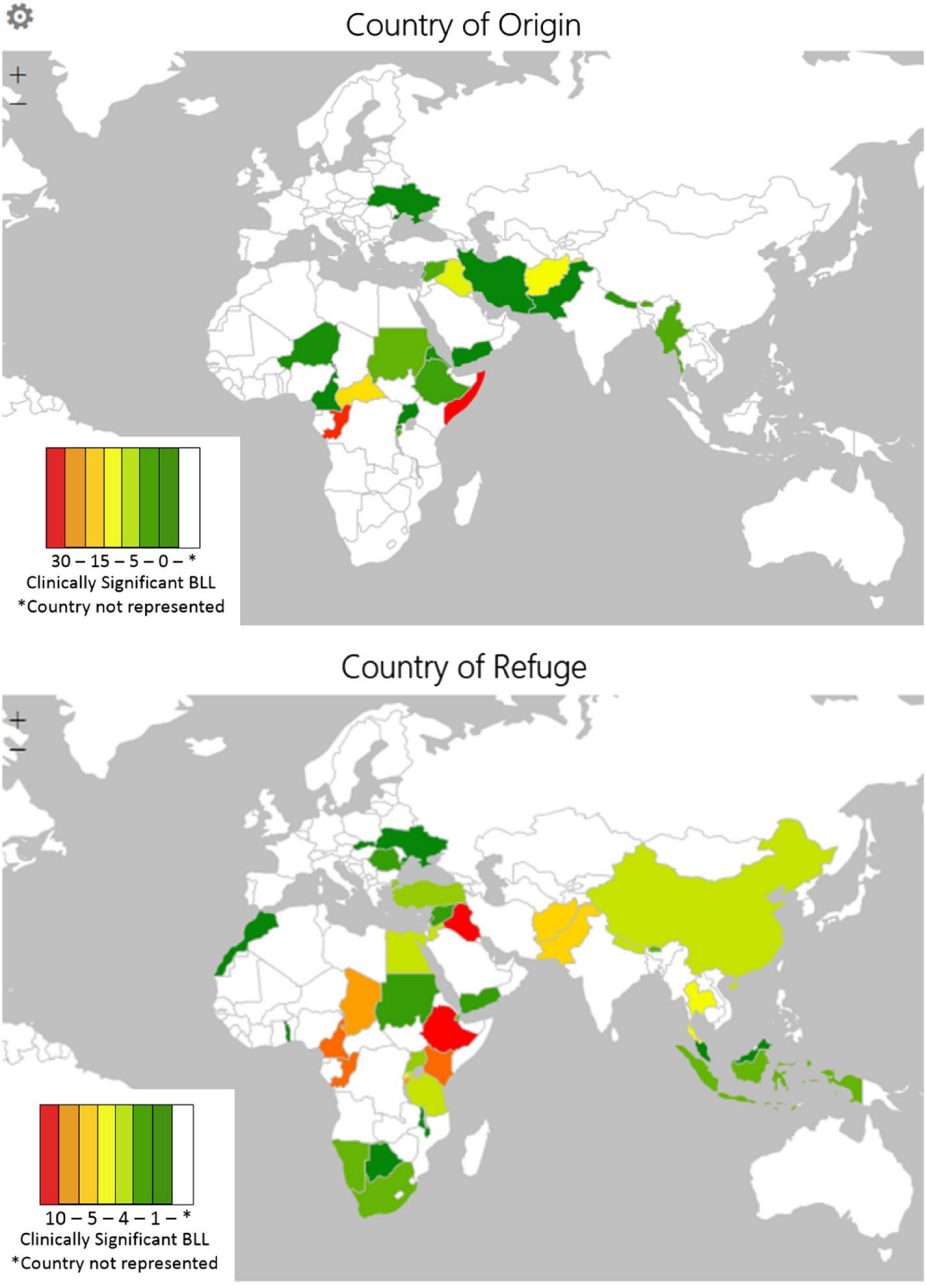
Total number of elevated BLLs by country of origin and refuge upon arrival

Figure 1 depicts the total number of elevated BLL (≥ 5 µg/dL) within each country of refuge upon arrival. The majority of the children with elevated BLL at baseline arrived in the US from countries in Africa (n = 66, 55.0%), with the remaining children with elevated levels hailing from countries within the Middle East (30.0%), Southeast Asia (14.2%), and Eastern Europe (0.8%). Table 2 shows BLLs greater than or equal to 5 µg/dL by country of refuge and origin upon arrival and follow-up and Table 3 depicts demographics and change in BLL if elevated upon arrival and/or follow-up of children hailing from Ethiopia.

**Table 2:** BLL above the reference level (BLL > or equal to 5 µg/dL) upon arrival and follow-up by country of refuge

Country of refuge			
Arrival		Follow-up	
Country	N (%)	Country	%
Ethiopia	10 (8.3)	Kenya	12 (17.1)
Iraq	10 (8.3)	Chad	8 (11.4)
Congo	8 (6.7)	Iraq	5 (7.1)
Cameroon	8 (6.7)	Ethiopia	4 (5.7)
Kenya	8 (6.7)	Tanzania	4 (5.7)
Burundi	7 (5.8)	Bhutan	3 (4.3)
Chad	7 (5.8)	Burundi	3 (4.3)
Afghanistan	6 (5.0)	Cameroon	3 (4.3)
Pakistan	6 (5.0)	Jordan	3 (4.3)
Rwanda	5 (4.2)	Nepal	3 (4.3)
Thailand	5 (4.2)	Thailand	3 (4.3)
China	4 (3.3)	Afghanistan	2 (2.9)
Egypt	4 (3.3)	Congo	2 (2.9)
Jordan	4 (3.3)	Djibouti	2 (2.9)
Nepal	4 (3.3)	Egypt	2 (2.9)
Tanzania	4 (3.3)	Rwanda	2 (2.9)
Turkey	3 (2.5)	Syria	2 (2.9)
Uganda	3 (2.5)	Uganda	2 (2.9)
Bhutan	2 (1.7)	China	1 (1.4)
Djibouti	2 (1.7)	Malta	1 (1.4)
Indonesia	2 (1.7)	Namibia	1 (1.4)
Namibia	2 (1.7)	Pakistan	1 (1.4)
S. Africa	2 (1.7)	Turkey	1 (1.4)
Romania	1 (0.8)		
Sudan	1 (0.8)		
Syria	1 (0.8)		
Yemen	1 (0.8)		

**Table 3:** Demographics of children arriving from Ethiopia: change in BLL if elevated upon arrival and/or follow-up

Study number	Family number	Country of refuge	Sex	Lead arrival ug/dL	Lead F/U ug/dL
Not elevated upon arrival, elevated at F/U					
93	54	Ethiopia	M	3	5
Elevated upon arrival, elevated at F/U					
644	342	Ethiopia	M	5	5
477	208	Ethiopia	F	6	5
437	190	Ethiopia	M	7	6
Elevated upon arrival, not elevated at F/U					
338	154	Ethiopia	M	5	4
490	211	Ethiopia	M	5	3
448	198	Ethiopia	F	5	3
476	208	Ethiopia	F	5	4
34	331	Ethiopia	M	5	3
677	267	Ethiopia	M	6	4
489	211	Ethiopia	M	6	2

Differences in risk upon arrival and post-resettlement were observed among male and female refugee children with respect to age. Younger females (0–4.9 years) were more likely to have elevated BLL upon arrival than older females (5–16 years) (28.4% vs. 12.0%, respectively; *p* < 0.01), while differences observed between age groups for males did not reach significance (23.7% (0–4.9 years) vs. 15.5% (5–16 years); *p* = 0.080). Again, younger females (0–4.9 years) were more likely than older females (5–16 years) to have elevated BLL at follow-up (20.0% vs. 4.9%, respectively; *p* < 0.01). Risk of elevated BLL at follow-up between males of different age groups was not significant (*p* = 0.067).

## Lead Levels After Resettlement

At repeat blood draw 3–6 months post-resettlement, 635 (90.1%) of children had BLLs below the reference level < 5 µg/dL, with 64 (9.1%) and 6 (0.9%) reporting elevated and highly elevated blood lead levels, respectively. The 70 children with elevated BLL at follow-up were 45.7% female with a mean age of 5.4 years (range 1–14 years). Younger children were more likely to have an elevated BLL at follow-up as compared to older children (16.0% 0–6 years vs. 5.8% 7–14 years; *p* < 0.01).

**Fig. 2:**
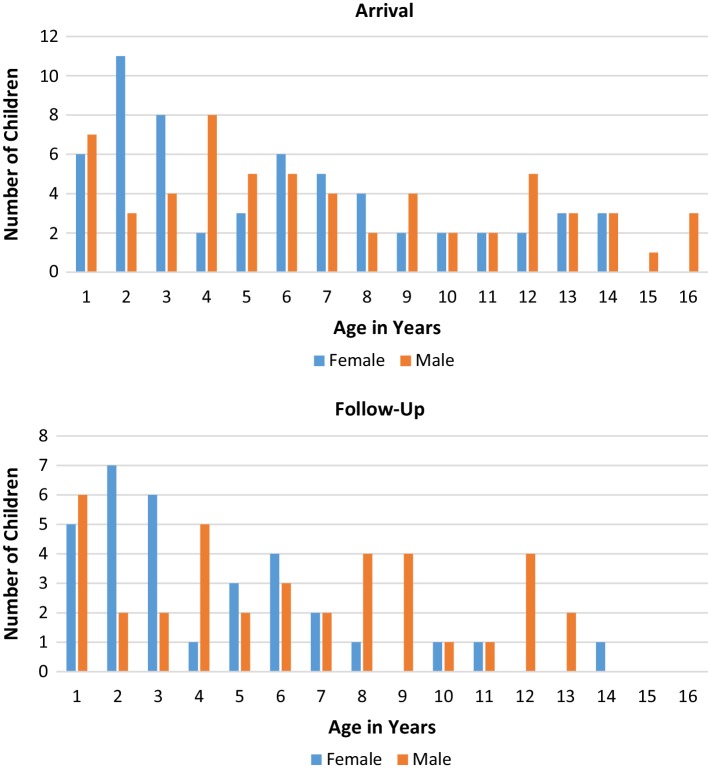
Distribution of children with a bll above the reference level upon arrival and follow-up by gender and age (years)

A total of 51 children were found to have BLL ≥ 5 µg/dL at both baseline and follow-up. The majority of these children (32, 62.7%) were from countries in Africa. Twenty-three out of the fifty-one children had BLLs at follow up that were equal to or higher than they were upon arrival. The average BLL increase in these 23 children was 1.56 µg/dL. Four of the new elevated exposures at follow up were increases of 2 µg/dL or greater (total range 0–17 µg/dL). No statistical differences were found between gender and elevated arrival BLL (p = 0.624) or gender and follow-up BLL (p = 0.333). Figure 2 shows the distribution of children with a BLL above the reference level upon arrival and follow up by gender and age (years).

## New Exposures in Syracuse

There were 19 (2.7%) children with normal BLL upon arrival and had a BLL over 5.0 µg/dL at follow-up. These 19 children were 42.1% female with a mean age of 5.63 years (range 1–13 years). Two of the families had multiple children with new exposures at follow up. (Family # 28 (2 of 6 children elevated) and # 147 (3 of 6 children elevated). The remaining 14 children were all from households with multiple children present but only had 1 individual affected. The data were also analyzed for any increase in BLL from baseline to follow-up. While not necessarily exceeding the CDC reference value, 211 (29.9%) children out of the 705 were found to have BLLs that increased from baseline to follow-up. The average BLL increase was 1.27 µg/dL.

### Analysis of Anemia on Arrival and Lead on Arrival and Follow-Up

**Fig. 3:**
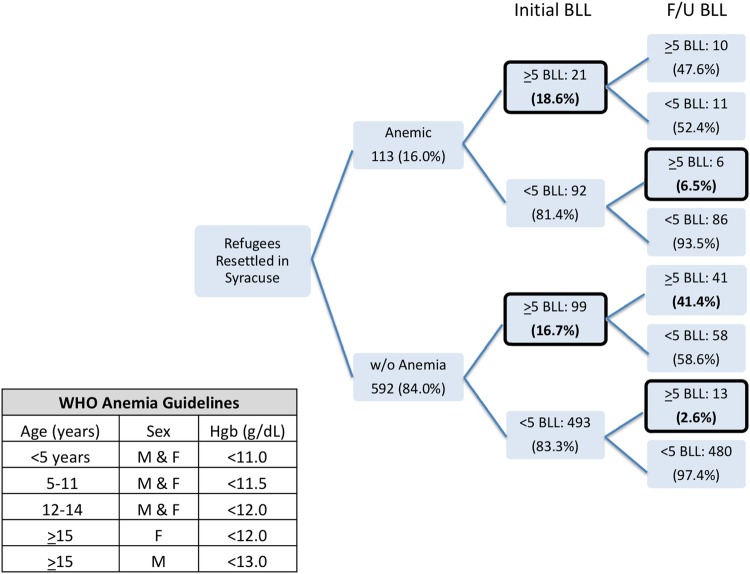
Anemia in the refugee population by lead level

There were 113 children (16.0%) who were anemic per WHO guidelines at arrival. As shown in Fig. 3, the number of refugees arriving with an elevated BLL did not differ significantly between refugees arriving with anemia (18.6%) and those without anemia (16.7%) (p = 0.630). However, of newly-arrived refugees initially screened to have a BLL below the reference level, a greater percentage of those with anemia had a BLL above the reference level on follow-up (6.5%) compared with those without anemia (2.6%) (p = 0.062 Fishers Exact Test). The majority of children with concurrent elevated BLL and anemia upon arrival were aged 1–5 years (14 of 21) and the remainder were between 6 and 15 years (7 of 21).

### Analysis of Red Cell Distribution Width Index (RDWI) and BLL

Analysis of RDWI among children with anemia revealed 24 children with indices less than 220, suggesting that other causes of anemia may be contributing to their low hemoglobin levels other than iron deficiency [9]. These 24 children represent those who may have microcytic anemia due to underlying hemoglobinopathy rather than iron deficiency. Of this subgroup of 24, 7 were found to have elevated lead levels upon arrival (29.0%); 6 out of the 7 children’s BLLs decreased by their follow-up blood draw.

## Discussion

### Consequences of Lead Exposure

Lead is a major environmental toxin and presents numerous health consequences for children [28]. No amount of lead exposure is normal nor safe, and researchers continue to identify harms at lower BLLs than previously thought dangerous [29]. BLLs under 10 µg/dL are associated with lower intelligence and social-behavioral concerns [30]. Elevated BLLs are associated with negative neurocognitive development as evidenced by lowering of IQ, impaired attention, learning, and memory, and aggression [31, 32]. Studies in adults have linked cumulative lead exposure with cognitive decline, irritability, headaches, joint pain, and gastrointestinal symptoms [32, 33]. Our study confirms prior literature showing refugee children are at risk for arriving with clinically significant lead exposure from their country of refuge and that resettlement does not eliminate risk of lead exposure.

### Existing Lead Prevention and Abatement Programming in Syracuse, NY

The burden of lead exposure remains significant in Syracuse, NY for all children regardless of refugee status [24]. The lead crisis in Syracuse is multifaceted and sustained through the combination of intense poverty, older housing stock, lack of laws to protect the community and diminished funding for lead abatement programs. One in ten children in the area have elevated BLLs and two of the worst cases of lead poisoning treated in 2018 were from refugee children [5, 34]. Although lead was banned from indoor paint in 1978, the median year of construction for more than half of the housing stock in the city of Syracuse was before 1939. Refugees are primarily settled in older, rental homes within the city. Some landlords are not compliant even after testing reveals that homes are contaminated with lead [35]. The U.S. Department of Housing and Urban Development (HUD) provided on average $2 million each year in funding to the city of Syracuse for lead abatement from 1994 to 2014. City grants were denied by HUD in 2014 and again in 2015 citing deficiencies in testing procedures and reporting errors in quarterly reports [36]. Onondaga County also receives federal grants for lead abatement but it is not enough to fill the gap left by the lack of funding for the city program and diminishing federal funding, overall. In addition, the city has declined to apply jointly for federal funding with the county. Local and federal government officials have expressed support for additional funding for lead abatement in Syracuse as a top priority, and HUD appears poised to reinstate funding for $4.1 million in the near future [36, 37].

### Refugee Children Found to have Exposure to Lead Post-Resettlement

Nearly 30% of refugee children included in this study were found to have increasing BLLs 3–6 months post-resettlement. While not all of these cases surpassed the reference level, the literature increasingly suggests that no amount of lead is safe [29]. Out of the 51 children with BLLs above the reference level upon arrival and follow-up, nearly half within this cohort were found to have higher levels at follow-up than arrival. This suggests that counseling regarding lead exposure (nutrition, behavior, etc.) upon arrival is not sufficient to keep children safe when families find themselves in homes that are not lead-safe. Primary prevention, through mitigation of risks in the home is paramount. However, non-compliant landlords [35] and loss of funding [38] are major barriers to lead abatement for the city’s many older rental homes.

Of additional concern, 3% of the children with normal BLL upon arrival were found to have a BLL above reference level at follow-up, suggesting that knowledge of risk as well as measures to mitigate risk in the community are lacking. Many of the children with new exposures hailed from countries within Africa. Additional cultural, religious, and/or value systems of families from these regions may need to be addressed to ensure that current US educational and protection efforts are translated across barriers to be optimally utilized. Access to resources for lead abatement programs may be difficult to navigate and financially inaccessible for refugee families who are placed in rental properties.

Although not a primary aim of this study, the role of anemia in refugee BLLs warrants further investigation with a larger cohort and additional behavioral and laboratory measures. The data suggest that children who were anemic upon arrival with sub-clinical BLLs were more likely to have a BLL above reference level upon follow-up than those without anemia.

## Implications

We need to continue to provide comprehensive services to this vulnerable population and identify additional risks such as iron deficiency that may increase their risks for lead exposure after arrival. We need to continue to support refugee families with the tools they need to communicate despite language barriers, to navigate our health system, to improve their health literacy so that these risks are minimized. Cultural humility is an approach we should take for all patients regardless of background, belief or value system, when we are explaining a diagnosis or engaging in shared decision making. This approach to care will undoubtedly improve health outcomes for this population and all children in the region.

Current screening guidelines for iron deficiency anemia and lead exposure in US-born children only focuses on children less than 2 years old undergoing rapid cognitive development and at risk of putting objects in their mouths [39]. These guidelines may limit the scope of the problem by not identifying all individuals at risk for lead poisoning and iron deficiency anemia, specifically menstruating adolescent females and women of childbearing age who are also at risk amidst unsafe living conditions. Due to the large number of refugees who left the Syracuse area during the study timeframe, secondary prevention through screening after the initial health assessment is also crucial to capture risk in migrating populations. These findings provide additional evidence supporting the dire need for primary prevention and screening through funding, policy and advocacy, which would remove or mitigate lead risks in housing for people of all ages and of all national origins.

## Limitations

Nearly 20% of the eligible cohort of refugee children did not have follow up lab tests to be included in this analysis, most commonly because they moved out of the area. Additionally, the clinical significance of a change in BLL by 1 µg/dL is unclear; however, all children in this study were followed by the same pediatric clinic and clinical laboratory to minimize sensitivity lost across different testing locations. All BLLs were obtained from a venous blood draw which is optimal over capillary blood draw which may have high rates of false positives. Even so, most laboratories can only achieve an error range of ± 2 µg/dL and federal guidelines allow for a range of ± 4 µg/dL [40]. These error rates are minimized by the use of the same laboratory over time, as was done in our sample. The associations between risk of lead exposure post-resettlement in children found to be iron deficient upon arrival and children who are not iron deficient must be explored further with a larger study cohort. In addition, the results of this study may not be generalizable to all pediatric refugees of other demographics, location, beliefs, and/or experiences.

## New Contribution to the Literature

Our study demonstrates a similar proportion of children arrive with lead exposure as reported elsewhere in the country but a greater risk of BLL rise after resettlement. Our data set is unique in that we were able to document both the country of origin and the family’s last site of refuge, which is most closely tied to their risks for lead exposure prior to resettlement. Our analysis of new exposures shows that while the majority of new exposures were < 5 years old, there remained a significant proportion that would have been missed on routine screening of 1 and 2 year old children in the U.S. It is important to remain aware of risk factors, such as iron deficiency in older children that also warrant lead screening. Ongoing efforts to address unsafe lead exposure through primary prevention (e.g. safe housing, lead abatement programs, education using translation where appropriate) will ultimately improve the health of all children locally, including resettled refugees.
